# Model-Guided Decision-Making for Thromboprophylaxis and Hospital-Acquired Thromboembolic Events Among Hospitalized Children and Adolescents: The CLOT Randomized Clinical Trial

**DOI:** 10.1001/jamanetworkopen.2023.37789

**Published:** 2023-10-13

**Authors:** Shannon C. Walker, Benjamin French, Ryan P. Moore, Henry J. Domenico, Jonathan P. Wanderer, Amanda S. Mixon, C. Buddy Creech, Daniel W. Byrne, Allison P. Wheeler

**Affiliations:** 1 Department of Pathology, Microbiology, and Immunology, Vanderbilt University Medical Center, Nashville, Tennessee; 2 Division of Pediatric Hematology/Oncology, Vanderbilt University Medical Center, Nashville, Tennessee; 3 Vanderbilt Vaccine Research Program, Vanderbilt University Medical Center, Nashville, Tennessee; 4 Department of Biostatistics, Vanderbilt University Medical Center, Nashville, Tennessee; 5 Department of Anesthesiology, Vanderbilt University Medical Center, Nashville, Tennessee; 6 Department of Biomedical Informatics, Vanderbilt University Medical Center, Nashville, Tennessee; 7 Department of Medicine, Vanderbilt University Medical Center, Nashville, Tennessee

## Abstract

**Importance:**

Rates of hospital-acquired venous thromboembolism (HA-VTE) are increasing among pediatric patients. Identifying at-risk patients for whom prophylactic interventions should be considered remains challenging.

**Objective:**

To determine whether use of a previously validated HA-VTE prognostic model, together with pediatric hematologist review, could reduce pediatric inpatient rates of HA-VTE.

**Design, Setting, and Participants:**

This pragmatic randomized clinical trial was performed from November 2, 2020, through January 31, 2022, at a single-center academic children’s hospital (Monroe Carell Jr Children’s Hospital at Vanderbilt). All pediatric hospital admissions (aged <22 years) under inpatient status were included and randomized.

**Intervention:**

All patients had an HA-VTE probability automatically calculated daily, which was visible to the hematology research team for patients in the intervention group. Patients with an elevated risk (predicted probability ≥2.5%) underwent additional medical record review by the research team to determine eligibility for thromboprophylaxis.

**Main Outcomes and Measures:**

The primary outcome was rate of HA-VTE. Secondary outcomes included rates of prophylactic anticoagulation and anticoagulation-associated bleeding events.

**Results:**

A total of 17 427 hospitalizations met eligibility criteria, were randomized, and were included in the primary analysis: patients had a median (IQR) age of 1.7 (0 to 11.1) years; there were 9143 (52.5%) female patients and 8284 (47.5%) male patients, and there were 445 (2.6%) Asian patients, 2739 (15.9%) Black patients, and 11 752 (67.4%) White patients. The 2 groups were evenly balanced in number (8717 in the intervention group and 8710 in the control group) and patient characteristics. A total of 58 patients (0.7%) in the control group and 77 (0.9%) in the intervention group developed HA-VTE (risk difference: 2.2 per 1000 patients; 95% CI, −0.4 to 4.8 per 1000 patients; *P* = .10). Recommendations to initiate thromboprophylaxis were accepted by primary clinical teams 25.8% of the time (74 of 287 hospitalizations). Minor bleeding events were rare among patients who received anticoagulation (3 of 74 [4.1%]), and no major bleeding events were observed during the study period. Among patients randomized to the control group, the model exhibited high discrimination accuracy (*C* statistic, 0.799, 95% CI, 0.725 to 0.856).

**Conclusions and Relevance:**

In this randomized clinical trial of the use of a HA-VTE prognostic model to reduce pediatric inpatient rates of HA-VTE, despite the use of an accurate and validated prognostic model for HA-VTE, there was substantial reluctance by primary clinical teams to initiate thromboprophylaxis as recommended. In this context, rates of HA-VTE between the control and intervention groups were not different. Future research is needed to identify improved strategies for prevention of HA-VTE and to overcome clinician concerns regarding thromboprophylaxis.

**Trial Registration:**

ClinicalTrials.gov Identifier: NCT04574895

## Key Points

**Question:**

Can an automated prognostic model embedded in the electronic medical record help prevent hospital-acquired venous thromboembolism (HA-VTE) among hospitalized children and adolescents?

**Findings:**

In this randomized clinical trial that included 17 427 pediatric hospitalizations, 58 patients (0.7%) in the control group and 77 (0.9%) in the intervention group developed HA-VTE, a non–statistically significant difference. Recommendations to initiate thromboprophylaxis were followed only 25.8% of the time.

**Meaning:**

Despite use of an accurate and validated prognostic model for HA-VTE, there was substantial reluctance by primary clinical teams to initiate thromboprophylaxis as recommended; in this context, HA-VTE rates between the control and intervention groups were not different.

## Teaser

This randomized clinical trial evaluates whether the use of a previously validated prognostic model for hospital-acquired venous thromboembolism (HA-VTE) reduces pediatric inpatient rates of HA-VTE.

## Introduction

Hospital-acquired venous thromboembolism (HA-VTE) is an increasing cause of morbidity and mortality among pediatric patients,^1,2^ and those who develop HA-VTE have longer hospital stays, increased medical costs, and increased risk of complications.^3,4,5^ Increasing effort has focused on early identification of pediatric patients at elevated risk for developing HA-VTE for whom thromboprophylaxis should be considered. Prior studies have sought to identify risk factors and develop prognostic models to assist in risk stratification,^6,7^ although many of these models are limited to specific patient subgroups and are not broadly applicable across all pediatric patients.^8,9,10^ Furthermore, to our knowledge, these models have not been prospectively assessed to determine their impact on patient outcomes in randomized controlled trials.

Knowing that prognostic models have been shown to better identify patients at elevated risk for HA-VTE than physician judgment alone,^11,12,13^ we previously developed and validated a prognostic model to identify general pediatric inpatients with elevated risk for developing HA-VTE.^14^ The model uses data available upon admission to estimate the probability of HA-VTE for each patient, which is updated every 24 hours as new data become available during hospitalization. For ease of use, the model is incorporated into the electronic medical record (EMR), such that variables are extracted and the predicted probability is calculated automatically without any need for clinician input of clinical or laboratory information. To assess the model’s impact on patient outcomes by identifying at-risk patients within a large general pediatric inpatient population, we conducted a randomized clinical trial using the HA-VTE prognostic model along with targeted hematology review of patients with elevated risk to determine whether the combination could decrease pediatric HA-VTE rates compared with usual care at our medical center.

## Methods

### Trial Design

The Children’s Likelihood of Thrombosis (CLOT) trial was an unblinded, pragmatic randomized clinical trial conducted at Monroe Carell Jr Children’s Hospital at Vanderbilt (MCJCHV) at Vanderbilt University Medical Center in Nashville, Tennessee.^15^ The trial was approved by the Vanderbilt University Medical Center Human Research Protections Program with waiver of informed consent due to minimal risk to participants and impracticality of obtaining informed consent from all admissions. The trial was registered with ClinicalTrials.gov prior to initiation of patient enrollment on September 28, 2020. The date of first patient enrollment was November 2, 2020; date of study completion, January 31, 2022; dates of data extraction and analysis, March 15, 2022, to February 17, 2023. Trial data were obtained from our institution’s EMR, including sociodemographic characteristics, medical history, and outcomes identified from *International Classification of Diseases, Ninth Revision *(*ICD-9*) and *International Statistical Classification of Diseases and Related Health Problems, Tenth Revision *(*ICD-10*) codes and medication orders (Supplement 1). This report follows the Consolidated Standards of Reporting Trials (CONSORT) reporting guidelines.

### Participants

Eligible participants were younger than 22 years and were admitted to a pediatric unit (ie, general pediatric wards; subspecialty wards; pediatric critical care units, including the pediatric intensive care unit and the pediatric cardiology intensive care unit; and the neonatal intensive care unit) at MCJCHV during the study period. Patients admitted under observation status, same-day surgery, or other admission categories were not eligible unless their status was changed to inpatient. Patients who were discharged or had a diagnosis of HA-VTE prior to randomization were excluded. Enrolled patients who were discharged from the hospital and subsequently readmitted during the study period remained eligible and were rerandomized. The primary analysis included all admissions during the study period, while a secondary sensitivity analysis included only the first admission for each patient.

### Randomization and Blinding

Upon inpatient admission orders being placed by the treating clinicians, eligible patients were randomized by an Epic BestPractice Advisory that ran in the background and was not seen by clinicians. The advisory programmatically generated a random binary value to determine whether the patient was assigned to the control group or intervention group. The group to which each patient was assigned was saved for that admission encounter and then extracted into a daily report.

The study principal investigator and the pediatric hematologists (S.C.W. and A.P.W.) who reviewed patients with elevated risk were unblinded to the intervention. Assignment to the intervention or control group was concealed for all other study personnel. Group assignments remained concealed from the biostatistics team and safety monitoring committee until primary data analysis was complete. The principal investigator was not responsible for diagnosing HA-VTE.

### Procedures

All pediatric patients admitted during the study period had their predicted probability of HA-VTE calculated^14^ on admission and daily thereafter (eMethods 1 in Supplement 2). For eligible patients randomized to the intervention group determined to be at elevated risk for developing HA-VTE (ie, predicted probability ≥2.5%), the hematologist performed a brief medical record review to determine whether the patient could benefit from pharmacologic thromboprophylaxis (with age- and weight-based dosing of unfractionated heparin or low-molecular-weight heparin [eMethods 1 in Supplement 2]) or whether the patient had known contraindications to anticoagulation. The hematologist then provided recommendations to the primary clinical team, including initiation of prophylactic pharmacologic anticoagulation when appropriate. The HA-VTE predicted probability value was not provided to the clinical teams; however, patient characteristics that contributed to their elevated predicted probability were discussed when requested. The recommendations were communicated verbally to the patient’s primary clinicians, either in person or via telephone, and documentation was placed in the EMR to indicate the patient was identified as being at elevated risk for developing HA-VTE. Whether the recommendation was accepted, and the reason for not accepting the recommendation, was captured.

Patients randomized to the control group received the current standard of care treatment; they were eligible for prophylactic pharmacologic initiation at the discretion of their clinical team, who would identify patients who may benefit and consult the pediatric hematology consult service for recommendations. There were no hospital-wide HA-VTE prophylaxis protocols in place during the study.

### Outcomes

The primary prespecified end point was an imaging-confirmed diagnosis of VTE during hospitalization (eMethods 2 in Supplement 2). Secondary outcomes included initiation of prophylactic anticoagulation, based on medication orders. To ensure patient safety, we assessed the frequency and severity of bleeding-related adverse events using the modified WHO bleeding scale among patients in the intervention group who initiated thromboprophylaxis.

### Statistical Analysis

The historical rate of HA-VTE at our institution was 10 per 1000 patients. With at least 15 000 patients randomized 1:1 to the intervention and control groups (ie, 7500 per group), a 2-sample Fisher exact test achieves 80% power to detect an absolute risk reduction from 10 per 1000 in the control group to 5.95 per 1000 in the intervention group with a type I error rate of 5%.

Primary analyses compared the intervention and control groups and were conducted by modified intention to treat, with the modification that randomized patients who already initiated thromboprophylaxis on the first day of their hospitalization were excluded. Secondary as-treated analyses were also conducted, considering whether treatment recommendations were followed by the primary clinical team. As-treated analyses were limited to the strata of patients with elevated risk of developing HA-VTE (predicted probability ≥2.5%), with comparisons between patients randomized to the control group, randomized to the intervention but treatment recommendations were not followed, and randomized to the intervention and treatment recommendations were followed.

Sociodemographic characteristics (including race and ethnicity as reported by patients and recorded in the EMR) and baseline clinical characteristics were summarized using standard descriptive statistics. Race and ethnicity were included to ensure the model performed consistently across all groups. The proportion of patients diagnosed with HA-VTE (primary outcome) and the proportion of patients who received prophylactic anticoagulation (secondary outcome) were compared between the intervention and control groups using unadjusted risk differences with 95% CIs. We did not specify a priori any adjustment variables to include in multivariable models, and no variable exhibited a clinically meaningful imbalance between the intervention and control groups. For the primary outcome, a 2-sided *P* < .05 (obtained from Pearson χ^2^ test) indicated statistical significance. Secondary subgroup analyses were performed by patient age, patient sex, patient race and ethnicity, admission type (emergency, newborn, elective, urgent, trauma), and risk of VTE at admission (predicted probability <2.5% or ≥2.5%). As-treated analyses were performed using logistic regression models that adjusted for the highest predicted probability of HA-VTE prior to diagnosis. Secondary and subgroup analyses were conducted without corrections for multiple comparisons and were therefore considered exploratory. Interim safety analyses were conducted every 6 months and reported to the safety monitoring committee, for which outcome rates were presented with group assignments concealed and without between-group statistical comparisons; therefore, no predetermined stopping rules were used. Performance of the prognostic model was summarized using the *C* statistic, sensitivity, and specificity; due to the potential for the model-guided intervention to impact outcomes for patients in the intervention group, these summaries were obtained for the control group only. All statistical analyses were prespecified and performed using reproducible research methods in R version 4.2.1 (R Project for Statistical Computing), including the R packages Hmisc and rms.

## Results

**Figure 1.**
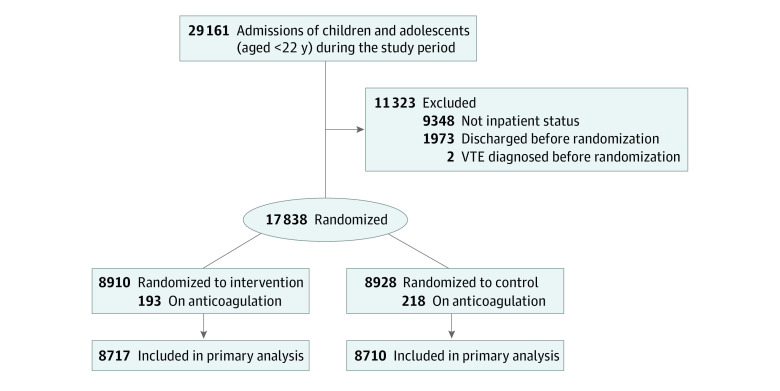
Participant Flow Through the Children’s Likelihood of Thrombosis Trial for Primary Intention-to-Treat Analysis

A total of 17 838 hospitalization admissions from 14 452 patients met eligibility criteria and were randomized (Figure 1): 8910 encounters were allocated to the intervention group and 8928 to the control group. Once patients were allocated into the intervention and control groups, hospitalizations during which patients were already receiving anticoagulation were identified through EMR orders, and these hospitalizations were excluded. The remaining 17 427 hospitalizations from 14 247 patients were included in the primary analysis. Patients had a median (IQR) age of 1.7 (0 to 11.1) years; there were 9143 (52.5%) female patients and 8284 (47.5%) male patients, and there were 445 (2.6%) Asian patients, 2739 (15.9%) Black patients, and 11 752 (67.4%) White patients. Patient characteristics were balanced across the groups in key demographic characteristics, including age, sex, race and ethnicity, insurance type, and the variables input to the prognostic model (Table 1). Among patients randomized to the control group, the model exhibited high discrimination accuracy (*C* statistic, 0.799; 95% CI, 0.725-0.856; sensitivity, 0.86; 95% CI, 0.75 to 0.94; specificity, 0.90; 95% CI, 0.89 to 0.90) (eTable 1 in Supplement 2).

**Table 1.** Characteristics of the Children and Adolescents Included in the Children’s Likelihood of Thrombosis Trial^a^

Characteristic	No. (%)	
	Control group (n = 8710)	Intervention group (n = 8717)
Age, median (IQR), y^b^	1.7 (0-11.0)	1.6 (0-11.2)
Sex		
Female	4174 (47.9)	4110 (47.1)
Male	4536 (52.1)	4607 (52.9)
Race^c^		
Asian	233 (2.6)	212 (2.4)
Black	1337 (15.4)	1402 (16.1)
Multiple races	257 (3.0)	237 (2.7)
Other^d^	26 (0.3)	32 (0.4)
White	5892 (67.6)	5860 (67.2)
Unknown^e^	975 (11.2)	974 (11.2)
Ethnicity^c^		
Hispanic	1248 (14.3)	1187 (13.6)
Non-Hispanic	7274 (83.5)	7322 (84.0)
Unknown^e^	188 (2.2)	208 (2.4)
Insurance type		
Government	5337 (61.3)	5316 (61.0)
Private	3259 (37.4)	3279 (37.6)
Other	28 (0.3)	15 (0.2)
Unknown^e^	86 (1.0)	107 (1.2)
History of cancer^b^	105 (1.2)	115 (1.3)
History of thrombosis^b^	14 (0.2)	14 (0.2)
Admission type		
Emergency	3879 (44.5)	3846 (44.1)
Newborn	2514 (28.9)	2496 (28.6)
Elective	1390 (16.0)	1416 (16.2)
Urgent	728 (8.4)	762 (8.7)
Trauma	194 (2.2)	193 (2.2)
Unknown^e^	5 (<0.1)	4 (<0.1)
Weekend or holiday admission^f^	3184 (36.6)	3218 (36.9)
Placement of a central line during admission^b^	846 (9.7)	888 (10.2)
Receipt of surgery during admission^b^	2331 (26.8)	2402 (27.6)
Blood gas performed during admission^b^	1580 (18.1)	1565 (18.0)
Cardiology consulted during admission^b^	1032 (11.8)	1013 (11.6)
Infectious disease consulted during admission^b^	1025 (11.8)	1060 (12.2)
Earliest lactate during admission, median (IQR), mmol/L^b^^,^^g^	1580 (18.1)	1565 (18.0)
Earliest MCHC during admission, median (IQR), g/dL^b^^,^^h^	33.7 (32.7-34.6)	33.7 (32.7-34.6)
Earliest RDW during admission, median (IQR), %^b^^,^^i^	13.9 (12.7-16.2)	13.9 (12.7-16.2)
Predicted probability of HA-VTE at admission, median (IQR), %	0.2 (0.1-0.4)	0.2 (0.1-0.4)
Length of hospital stay, median (IQR), d	2.7 (1.8-5.1)	2.7 (1.8-5.2)

Abbreviations: HA-VTE, hospital-acquired venous thromboembolism; MCHC, mean corpuscular hemoglobin concentration; RDW, red blood cell distribution width.

SI conversion factors: To convert lactate to milligrams per deciliter, divide by 0.111; to convert MCHC to grams per liter, multiply by 10.

^a^ Includes all admissions during the study period.

^b^ Included as an input to the prognostic model for hospital-acquired venous thromboembolism.^14^

^c^ Reported by patients and recorded in the electronic medical record.

^d^ Includes American Indian and Native Hawaiian.

^e^ Not recorded in electronic medical record.

^f^ Weekend admission defined as admission occurring at any time on a Friday, Saturday, or Sunday. Non–weekend holidays were Christmas, Thanksgiving, and New Year’s Day.

^g^ Unknown for 11 338 patients (65.1%). For calculation of the predicted probability of hospital-acquired venous thromboembolism, unknown values were replaced with the value 1.3 mmol/L.

^h^ Unknown for 5292 patients (30.4%). For calculation of the predicted probability of hospital-acquired venous thromboembolism, unknown values were replaced with the value 34.0 g/dL.

^i^ Unknown for 5301 patients (30.4%). For calculation of the predicted probability of hospital-acquired venous thromboembolism, unknown values were replaced with the value 14.3%.

Among 8710 hospitalizations in the control group, 58 (0.7%) experienced HA-VTE; among the 8717 hospitalizations in the intervention group, 77 (0.9%) experienced HA-VTE (risk difference, 2.2 per 1000 patients, 95% CI, −0.4 to 4.8 per 100 patients, *P* = .10) (Table 2). The location of these HA-VTEs is described in eTable 2 in Supplement 2. Of the 58 hospitalizations with HA-VTE in the control group, 50 (86.2%) were identified as being at elevated risk by having a predicted probability of 2.5% or greater during their hospitalization; similarly, in the intervention group, 71 of 77 patients (92.2%) with HA-VTE had a predicted probability of 2.5% or greater. Of the 14 patients who were diagnosed with HA-VTE and did not have a predicted probability of 2.5% or greater during their hospitalization, additional medical record review revealed that 10 HA-VTEs were diagnosed as incidental findings. The additional 4 HA-VTEs were diagnosed in patients younger than 8 months. The number of patients receiving thromboprophylaxis in the control group was 154 (1.8%) and the number in the intervention group was 231 (2.7%), with a risk difference of 8.8 per 1000 patients (95% CI, 4.5 to 13.2 per 1000 patients).

**Table 2.** Primary and Secondary Outcomes for All Randomized Patients^a^

Outcome	Patients, No. (%)		Risk difference per 1000 patients (95% CI)
	Control group (n = 8710)	Intervention group (n = 8717)	
Primary outcome			
HA-VTE	58 (0.7)	77 (0.9)	2.2 (−0.4 to 4.8)^b^
Secondary outcome			
Prophylactic anticoagulation	154 (1.8)	231 (2.7)	8.8 (4.5 to 13.2)
Post hoc secondary outcomes			
All-cause in-hospital mortality	132 (1.5)	123 (1.4)	−1.0 (−4.6 to 2.5)
Died with an HA-VTE	10 (0.1)	11 (0.1)	0.1 (−0.9 to 1.1)
30-d Readmission	845 (9.7)	788 (9.0)	−6.6 (−15.3 to 2.0)

Abbreviation: HA-VTE, hospital-acquired venous thromboembolism.

^a^ Includes all admissions during the study period.

^b^ *P* = .10.

**Figure 2.**
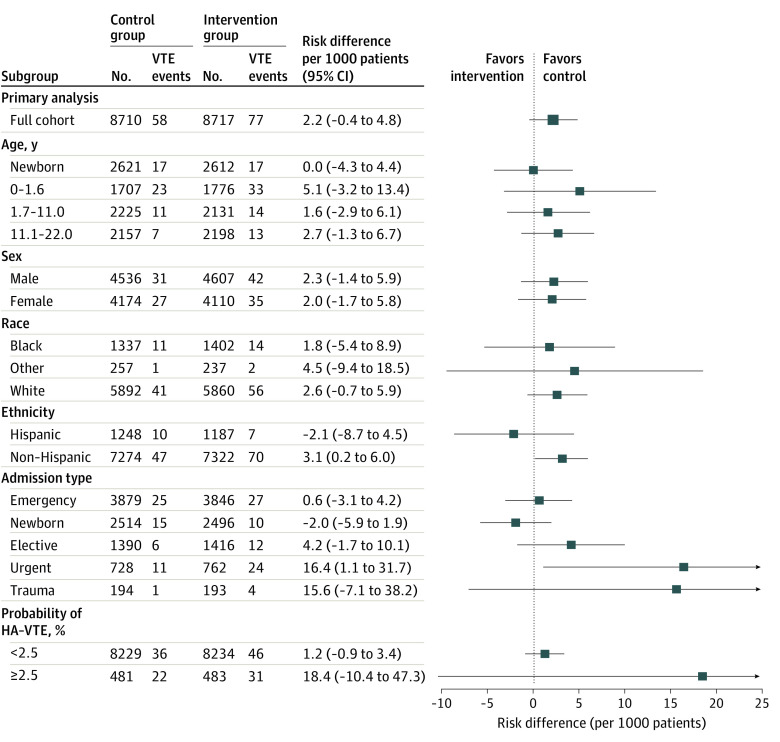
Rate of Hospital-Acquired Venous Thromboembolism (HA-VTE) Among Subgroups of Patients Other race includes American Indian, Asian, multiple races, and Native Hawaiian. Probability of HA-VTE calculated at admission.

Secondary sensitivity analyses limited to the first admission during the study period, for which 7127 patients were randomized to the control group and 7120 were randomized to the intervention group, provided similar results (eTables 3 and 4 in Supplement 2). Secondary subgroup analyses were performed by age, sex, race and ethnicity, admission type, and risk of VTE at admission (predicted probability <2.5% or ≥2.5%); these analyses are shown in Figure 2. No clinically meaningful differences were observed in efficacy of the intervention across these groups.

Within the control and intervention groups, hospitalizations were separated into those patients with elevated risk of developing HA-VTE (predicted probability ≥2.5%) and those without elevated risk (eFigure in Supplement 2). The number of patients with elevated risk was similar with 932 (10.7%) in the control group and 974 (11.2%) in the intervention group. For practical reasons, patients in the intervention group were reviewed daily on weekdays, but not on weekends or holidays; therefore, 490 patients in the intervention group with elevated risk of developing HA-VTE were reviewed by the research team. Of those, 203 patients were identified by the research team as not being candidates for prophylactic pharmacologic intervention due to significant prematurity (<34 weeks), upcoming extensive surgical procedure, kidney impairment, expected discharge within 24 hours, or increased bleeding risk (eTable 5 in Supplement 2). The research team recommended that the remaining 287 patients be treated with prophylactic anticoagulation, which was initiated during 74 hospitalizations (25.8%), and in the remaining 213 hospitalizations (74.2%), prophylactic anticoagulation was not initiated by the primary team. The reasons that the primary teams cited when not initiating recommended prophylaxis included imminent patient discharge, likely central line removal within 24 hours, and a consulting subspecialist declined the recommendation (eTable 6 in Supplement 2).

We compared the rate of HA-VTE among 3 groups of patients with elevated risk (predicted probability ≥2.5%): those randomized to the control group (50 HA-VTE in 932 hospitalizations [5.4%]), randomized to the intervention but treatment recommendations were not followed (9 of 213 [4.2%]), and randomized to the intervention and treatment recommendations were followed (7 of 74 [9.5%]) (Table 3; eTable 7 in Supplement 2). The median (IQR) highest predicted probability prior to developing HA-VTE was 6.3% (3.4%-11.2%) in the control group, 7.9% (4.4%-11.8%) in the intervention group for whom recommendations for thromboprophylaxis were not followed, and 9.0% (6.2%-13.6%) in the intervention group for whom recommendations for thromboprophylaxis were followed. After adjustment for predicted probability of HA-VTE, there was no difference in the rate of HA-VTE between these groups (recommended and not initiated vs control: adjusted odds ratio, 0.84; 95% CI, 0.38-1.67; recommended and initiated vs control: adjusted odds ratio, 1.58; 95% CI, 0.62-3.50).

**Table 3.** Primary and Secondary Outcomes for Patients With Elevated Risk for Developing HA-VTE^a^

Outcome	Patients, No. (%)		
	Control group (n = 932)	Recommended and not initiated (n = 213)	Recommended and initiated (n = 74)
Primary outcome			
HA-VTE^b^	50 (5.4)	9 (4.2)	7 (9.5)
Secondary outcome			
Bleeding severity^c^			
Grade 1 (minor)	Not assessed	Not assessed	1 (1.4)
Grade 2 (minor)	Not assessed	Not assessed	2 (2.7)
Grade 3 (major)	Not assessed	Not assessed	0
Grade 4 (major)	Not assessed	Not assessed	0
Post hoc secondary outcomes			
All-cause in-hospital mortality	65 (7.0)	9 (4.2)	5 (6.8)
Died with an HA-VTE	10 (1.1)	2 (0.9)	1 (1.4)
30-d Readmission	220 (23.6)	64 (30.0)	13 (17.6)

Abbreviation: HA-VTE, hospital-acquired venous thromboembolism.

^a^ Elevated risk defined as a predicted probability of developing hospital-acquired venous thromboembolism of 2.5% or greater.

^b^ After adjustment for the predicted probability of HA-VTE (eTable 7 in Supplement 2), there was no difference in the rate of HA-VTE between these groups.

^c^ Modified World Health Organization bleeding scale, assessed only in patients who were randomized to the intervention group, treatment was recommended, and treatment recommendations were followed.

Once patients were initiated on prophylactic anticoagulation per the research team recommendations, they were followed up via medical record review and regular discussions with the primary team to assess for bleeding. Only intervention group patients receiving anticoagulation had bleeding scores tracked and captured due to the real-time nature of the study design. Three patients developed minor bleeding: 1 developed minor oropharyngeal bleeding (World Health Organization [WHO] bleeding scale grade I), 1 developed hemoptysis (WHO bleeding scale grade II), and 1 developed bleeding from procedure sites (WHO bleeding scale grade II). All 3 patients had resolution of the bleeding upon cessation of anticoagulation. No patients developed major bleeding events during the study (WHO bleeding scale grade III or IV).

## Discussion

The development of pediatric HA-VTE is associated with longer hospital stays, increased medical costs, and subsequent medical complications,^3,4,5^ and improving outcomes in pediatric patients identified to be at risk for HA-VTE has been challenging.^11,12,13^ The innovative study design of the CLOT trial used an automated EMR-based clinical decision support tool to calculate the probability of a patient developing HA-VTE and included integration into the EMR for key functions, including automated patient-level randomization, which led to large, well-balanced groups; automated implementation of the prognostic model to easily identify at-risk patients; and automated data extraction, which facilitated daily updates as patients’ clinical scenario evolved. The HA-VTE prognostic model has been shown to accurately identify at-risk patients in derivation and validation cohorts^14^ as well as among patients included in this trial; however, incorporating the model into routine clinical practice did not decrease HA-VTE rates. We postulate that this is chiefly due to low rates of thromboprophylaxis being initiated when recommended. Additionally, the study did not identify any episodes of severe bleeding in patients who received thromboprophylaxis.

We hypothesize that the intervention’s primary lack of success in decreasing HA-VTE rates was due to low rates of thromboprophylaxis acceptance when recommended. Multiple clinical teams cited their concern that initiating pharmacologic prophylaxis would increase their patient’s risk of bleeding; however, prior literature^16,17,18^ and the safety work performed during this trial demonstrate that it is unlikely that appropriately recommended prophylactic anticoagulation will increase an individual’s risk for significant bleeding. Further work is planned to identify potential barriers as well as to optimize the implementation of incorporating the model into clinical practice, with the hope of improving acceptance of the hematology recommendations during future trials.

Other reasons that the intervention may have been unsuccessful include potentially ineffective dosing of thromboprophylaxis. During the study period, we recommended standard weight- and age-based prophylaxis dosing of unfractionated heparin and low-molecular-weight heparin for elevated risk patients deemed clinically safe for pharmacologic prophylaxis; however, recent data have emerged suggesting that HA-VTE associated with a central venous line (CVL) in patients with a history of HA-VTE may be prevented more effectively by using higher doses of pharmacologic prophylaxis.^19^ This could contribute to the overall lack of change in HA-VTE rates despite an increased number of patients who received thromboprophylaxis in the intervention group. In addition, by identifying patients with elevated risk, primary teams may have been motivated to make nonpharmacologic changes to decrease a patient’s risk for HA-VTE, such as removing a CVL sooner or using increased mobilization, rather than initiating pharmacologic prophylaxis. While these nonpharmacologic interventions may have decreased a patient’s risk for HA-VTE, this risk reduction is unable to be quantified and likely contributes to the lack of difference in HA-VTE rates seen in the study.

### Strengths and Limitations

Despite the observed lack of efficacy in this study, strengths of the study include its originality as, to our knowledge, the first large pediatric study to demonstrate the use of a randomization tool integrated into the EMR to provide unbiased, automated randomization for more 17 000 patients. Furthermore, the model was updated with new EMR information daily throughout each patient’s admission, which may contribute to the slightly different *C* statistic values compared with the original model.^14^ Using a pediatric hematologist to make the thromboprophylaxis recommendations as well as notifying the clinical teams of these recommendations allowed clinician concerns to be discussed directly. Clinicians and clinical teams often voiced strong acceptance or opposition to thromboprophylaxis recommendations; thus, using patient-level randomization avoided clinician or unit-dependent biases. The study also demonstrates the feasibility of performing a large-scale randomized trial without the need for significant external funding. Using readily available data that updates frequently to identify at-risk patients within a large general population that may benefit from subspecialist expertise without the need for clinical identification of risk can hopefully reduce the number of patients who experience potentially preventable complications.

We acknowledge the following limitations. First, although randomization and calculation of HA-VTE risk was automated in the EMR, subsequent manual review by the study team was only performed on weekdays; therefore, patients were randomized to the intervention who had elevated risk but were not reviewed. These patients were included in the intervention group in the primary intention-to-treat analysis but were not included in the as-treated analysis; both analyses failed to show benefit of the intervention. Second, while this study was only performed at a single site, we have successfully demonstrated the ability of an automated prognostic model incorporated into the EMR to accurately identify patients at elevated risk, which we are actively working to expand to additional academic children’s hospitals, which may have different baseline rates of HA-VTE due to demographic variability and different strategies for pharmacologic thromboprophylaxis, mobilization, and CVL removal.

## Conclusions

In this randomized clinical trial evaluating the use of a real-time, prognostic model to reduce rates of HA-VTE in hospitalized children and adolescents, the automated, EMR-integrated model functioned well to estimate risk of pediatric inpatients developing HA-VTE and did not demonstrate an increased risk of bleeding, but overall acceptance of the recommendations from the hematology research team was low and use of the model in this context did not improve patient outcomes. Moving forward, we are considering additional ways to assess the success of future studies, including the use of quality improvement metrics such as aim statements, plan-do-study-act cycles, and run charts, in addition to more traditional research mechanisms to identify the best strategies to prevent pediatric HA-VTEs. Overall, we anticipate optimizing anticoagulation dosing and overcoming barriers to implementation and adoption of the HA-VTE model in clinical practice to be the most important next steps.

## Notes

Supplement 1.
Trial Protocol

Supplement 2. **eMethods 1. **Details on Study Procedures **eMethods 2. **Details on Study Outcomes **eTable 1.** Confusion Matrix for the Prognostic Model at a Cut Point of 2.5% **eTable 2. **Type of Thromboembolism **eTable 3. **Characteristics of the Children and Adolescents Included in the CLOT Trial for Their First Admission During the Study Period **eTable 4. **Primary and Secondary Outcomes for All Randomized Patients for Their First Admission During the Study Period **eTable 5. **Reasons for Not Recommending Initiation of Pharmacologic Thromboprophylaxis to the Primary Team for Patients Randomized to the Intervention and With Elevated Risk for Developing Hospital-Acquired Venous Thromboembolism **eTable 6. **Reasons That the Primary Team Rejected the Recommendation for Initiating Pharmacologic Thromboprophylaxis for Patients Randomized to the Intervention and With Elevated Risk for Developing Hospital-Acquired Venous Thromboembolism **eTable 7. **Characteristics of the Children and Adolescents Included in the CLOT Trial with Elevated Risk for Developing Hospital-Acquired Venous Thromboembolism **eFigure. **Flow of Participants Through the CLOT Trial for Secondary As-Treated Analysis

Supplement 3.
Data Sharing Statement
