# Supplementary material for: Model-Guided Decision-Making for Thromboprophylaxis and Hospital-Acquired Thromboembolic Events Among Hospitalized Children and Adolescents: The CLOT Randomized Clinical Trial
Source: JAMA Netw Open. 2023 Oct 13;6(10):e2337789. doi: 10.1001/jamanetworkopen.2023.37789 (PMC10576217; doi:10.1001/jamanetworkopen.2023.37789)
Supplement: Supplement 1. — Trial Protocol [file jamanetwopen-e2337789-s001.pdf]

Using a Real-Time Risk Prediction Model to Identify Pediatric Patients at Risk for Thrombotic Events

Shannon Walker, MD
Clinical Fellow, PGY-7
Department of Pathology, Microbiology, and Immunology
Vanderbilt University Medical Center

Clinical Mentors:
Allison Wheeler, MD, MSCI, Assistant Professor
Department of Pathology, Microbiology, and Immunology and Department of Pediatric Hematology/Oncology
Vanderbilt University Medical Center

C. Buddy Creech, MD, MPH, Associate Professor
Vanderbilt Vaccine Research Program and Division of Pediatric Infectious Diseases
Vanderbilt University Medical Center

Biostatisticians:
Dan W. Byrne, MS
Department of Biostatistics
Vanderbilt University Medical Center

Ryan Moore, MS
Department of Biostatistics
Vanderbilt University Medical Center

Benjamin French, PhD
Department of Biostatistics
Vanderbilt University Medical Center

Protocol Version: 1.2
Protocol Date: March 7, 2022

Table of Contents

Table of Contents 2
1 Study Team Contact Information 3
2 Background 3
2.1 Introduction 3
2.2 Model Development 4
3 Rationale and Specific Aims 4
4 Eligibility Criteria 4
5 Enrollment/Randomization 5
5.1 Enrollment 5
5.2 Randomization 5
6 Study Procedures 5
6.1 Intervention and Control Arms 5
6.2 Intervention Arm 5
6.3 Control Arm 6
6.4 Outcomes 6
6.5 Primary Outcomes 6
6.6 Secondary Outcomes 7
7 Risks of Intervention 7
8 Reporting of Adverse Events or Unanticipated Problems Involving Risk to Participants or Others 8
8.1 Definition of Adverse Event 8
8.2 Definition of Serious Adverse Event 8
8.3 Reporting of SAE 9
8.4 Recording of AEs/SAEs 9
8.5 Safety Monitoring Committee 9
9 Study Withdrawal/Discontinuation 10
10 Statistical Considerations 10
10.1 Statistical Analysis Plan 10
10.2 Sample Size Considerations 10
11 Privacy/Confidentiality Issues 11
12 Follow-up and Record Retention 11
13 References 12

1 Study Team Contact Information

Shannon Walker, MD
Preston Research Building #397
2200 Pierce Ave, Nashville TN 37232
Shannon.walker@vumc.org
Desk: 615-936-5893, Work cell: 615-939-6976

Allison Wheeler, MD, MSCI
Preston Research Building #397
2200 Pierce Ave, Nashville TN 37232
Allison.p.wheeler@vumc.org
Desk: 615-875-2197, Work cell: 508-868-5839

C. Buddy Creech, MD, MPH
Vanderbilt Vaccine Research Program
CCC-5323 Medical Center North
1211 Medical Center Drive, Nashville TN 32732
Buddy.creech@vumc.org
Phone: 615-343-2877

2 Background

2.1 Introduction

Hospital acquired venous thromboembolism (VTE) has been identified as an increasing cause of morbidity and mortality in pediatric populations. While overall a rare complication in children, annual rates have been increasing, with a 70% increase in pediatric VTE demonstrated from 2001 to 2007[1]. Despite the increasing incidence, many treatment recommendations for pediatric VTE have been extrapolated from adult data[2]. Because children with VTE experience longer hospital stays, increased medical costs[3], and subsequent medical complications[4,5], it is important to identify patients early who are at increased risk. Given these risks, it is imperative that we develop a more personalized approach to identify children at highest risk for VTE so that focused interventions can be initiated.

Prior risk prediction models have been developed to evaluate pediatric patients at risk for VTE development[6,7]. These models, however, suffer from small samples sizes (<1000 patient observations) and typically are not prospectively validated[8,9]. Since risk prediction models have been shown to identify patients at high-risk for VTE events

better than physician judgement alone[10], our goal is to develop and validate a predictive model of pediatric thrombosis using a large, single center, retrospective cohort.

2.2 Model Development

We evaluated data from 111,352 admissions to Vanderbilt Children's Hospital from 1/1/2010 to 10/31/2017. Cases were identified from ICD9/10 codes and a subset of these records were reviewed for accuracy. Potential covariates were identified from known pediatric risk factors for VTE development prior to data collection, and the corresponding clinical data were electronically extracted through the Vanderbilt Research Derivative. Logistic regression was used to test the association of potential risk factors with development of VTE. Variable inclusion in the final model was based on data reduction methods including univariate analysis significance, availability in routine medical records, clinical expertise, and assessment of collinearity between variables. The model was validated using an internal bootstrap calibration.

We identified 110,537 controls and 815 cases within the cohort. The most significant variables for developing VTE were a history of thrombosis (OR 8.7, 95% CI 6.6 –11.3, $p<0.01$), presence of a central venous line (OR 4.9, 95% CI 4.0 – 5.8, $p<0.01$), and cardiology consultation (OR 4.0, 95% CI 3.3 – 4.8, $p<0.01$). Additional significant variables included whether a blood gas was performed, whether the patient had an infectious diseases consultation, patient age, mean corpuscular hemoglobin concentration (MCHC), red cell distribution width (RDW), and lactate. We selected these 11 final variables for use in the model, which yielded excellent discriminatory power (C-statistic = 0.907 in the model development cohort).

3 Rationale and Specific Aims

We have developed a general electronic health record (EHR)-based pediatric risk prediction model that can identify pediatric patients upon admission who are at higher than average risk for developing a VTE. We plan to evaluate whether using this VTE model risk score increases the number of high-risk patients screened by the VTE research team. The VTE research team will then consider various clinical recommendations, including potential prophylactic anticoagulation. We hypothesis that using this clinical support tool will lead to an overall reduction in the number of pediatric VTE seen in our institution.

4 Eligibility Criteria

Inclusion criteria:

1. All pediatric patients 0-21 years of age who are admitted to an inpatient unit of Vanderbilt Children's Hospital (VCH) will be included in the study.

Subjects must not meet any of the following exclusion criteria:

1. Receiving prophylactic or therapeutic dosing of anticoagulants, including enoxaparin, warfarin, bivalirudin, apixaban, rivaroxaban, dabigatran, and edoxaban.
2. Patients admitted under "observation status"

5 Enrollment/Randomization

5.1 Enrollment

All patients admitted to the VCH during the study period will be enrolled if they meet the eligibility criteria listed above. We are requesting waiver of consent for this trial due to the impracticality of obtaining consent for every pediatric patient admitted to VCH and overall minimal risk to participants.

5.2 Randomization

Patients will be randomized 1:1 to the intervention or control arms upon admission via an automatic process built into the electronic health record. Both arms will continue to receive local standard of care practice for VTE prophylaxis.

6 Study Procedures

6.1 Intervention and Control Arms

Patients will be enrolled into the study upon admission to Vanderbilt Children's Hospital. All patients in both arms will have a VTE risk percentage calculated based on their values in the logistic regression model. This will initially be calculated at the time of admission and only be available in real-time to the study team for the intervention arm.

6.2 Intervention Arm

Patients in the intervention arm will have their VTE risk prediction scores presented to the study team daily on weekdays via Epic report, which will list patients in descending order of risk severity for review by the VTE research team each weekday. Starting with the highest risk patients, the VTE research team will review each patient and clinical situation, and then the VTE research team will directly discuss risks/benefits

of prophylactic anticoagulation with the admitting team. Patients with a risk score <2.5% will not be reviewed, and we anticipate most of the intervention arm patients will fall into this category (based on our previous data, we anticipate >90% of all patients will score <2.5%). The VTE risk report will be re-calculated based on updated EHR data every day at midnight.

6.3 Control Arm

Patients randomized to the control arm will continue to receive Vanderbilt Children's Hospital standard of care anticoagulation practice, which is at the discretion of the admitting team. In general, nearly no pediatric patients are offered prophylactic anticoagulation unless a previous VTE has been identified. This currently is at the discretion of the provider and no risk scoring is used. VTE risk prediction scores will be calculated and stored for analysis, these will not be visible to the study team in real time.

6.4 Outcomes

6.5 Primary Outcomes

The primary outcome will be to determine whether the number of VTE events is lower in the intervention arm than the control arm. The endpoint measure will be calculated as the frequency of VTE events per hospital admission encounter per arm.

VTE outcomes will be tracked in both arms of the study on a monthly basis. The radiology coordinator will provide a report of all pediatric radiology studies completed in the prior month. The VTE research team will filter the data based on the location that the imaging was performed (such as inpatient at VCH) and type of radiology study to best identify VTEs (such as ultrasounds with doppler and CT-angiograms). We will then review the filtered report to identify the MRN of patients and the date of the VTEs that were identified during that time period.

An additional data sample will be obtained from the EMR one month after enrollment completion to assess ICD9/ICD10 codes for acute VTE (utilizing codes established during model development and validation) during the trial period to further confirm all VTE events were appropriately captured in the monthly reports.

6.6 Secondary Outcomes

Secondary outcomes will include:

1. Total number of patients, without contraindications to anticoagulant medications as described by the prescriber information for heparin and enoxaparin determined by the consulting hematologist, who are begun on prophylactic anticoagulation, by study arm
2. Total number of high-risk patients, without contraindications to anticoagulant medications as described by the prescriber information for heparin and enoxaparin determined by the consulting hematologist, who are begun on prophylactic anticoagulation, by study arm
3. Total number of patients, without contraindications to anticoagulant medications as described by the prescriber information for heparin and enoxaparin determined by the consulting hematologist, who are begun on anticoagulation medications compared to the total number of patients for which initiation of anticoagulation was recommended by the VTE research team.
4. Frequency of bleeding-related adverse events per number of patients begun on prophylactic anticoagulation, scored using the modified WHO bleeding scale, by study arm, during hospitalization

7 Risks of Intervention

The primary focus of our study is to determine if a VTE risk prediction tool increases the number of high-risk patients reviewed by the pediatric VTE research team. The majority of the patients in the study will be considered low risk (based on previous data, we anticipate >90% of all patients to fall into this category). High-risk patients in the intervention arm will receive an additional chart review and subspecialty expertise. Part of that subspecialty expertise may include recommendations to initiate prophylactic anticoagulation. The use of pharmacologic anticoagulation is the standard of care among peer pediatric institutions and among adult hospitalized patients (including at Vanderbilt University Medical Center). In fact, nearly all adult patients are provided some type of VTE prophylaxis upon admission. The decision to start patients on prophylactic anticoagulation is complex and involves consideration of the patient’s clinical status and review for possible contraindications. We would not consider patients for anticoagulation with severe bleeding disorders, clinically significant hemorrhage, severe renal dysfunction, or anything in the opinion of the investigator that would jeopardize the safety of the subject. Pharmacologic prophylaxis is performed using various anticoagulant medications, most commonly with heparin or enoxaparin.

These drugs are commonly used, FDA-approved for anticoagulation, and considered to be safe; side effects of these medications include injection site reactions, bleeding, and rarely, thrombocytopenia. In several studies of pediatric VTE pharmacologic prophylaxis, there were very few bleeding episodes reported[11,12,13].

8 Reporting of Adverse Events or Unanticipated Problems Involving Risk to Participants or Others

8.1 Definition of Adverse Event

An Adverse Event (AE) means any untoward medical occurrence associated with the use of an intervention in humans, whether or not considered intervention related. An AE can therefore be any unfavorable and unintended sign (including an abnormal laboratory finding), symptom, or disease temporally associated with the use of a medicinal (investigational) product. Any medical condition present at the time of enrollment will be considered as baseline and not reported as an AE.

Table 1

WHO bleeding scale

Grade 0	No bleeding
Grade 1	Petechial bleed
Grade 2	Mild blood loss (clinically significant)
Grade 3	Gross blood loss requires transfusion (severe)
Grade 4	Debilitating blood loss, retinal/cerebral bleed (associated with fatality)

Due to the nature of the study, which is being performed to evaluate a clinical support tool, we anticipate minimal AEs. Due to the possible clinical recommendation for anticoagulation, we are planning to only collect coagulation/hemostasis-related AE’s in the study. We will use the WHO bleeding scale to grade any bleeding events (table 1).

8.2 Definition of Serious Adverse Event

An AE or suspected adverse reaction is considered serious (an SAE) if, in the view of the investigator, it results in any of the following outcomes: death, a life-threatening AE, substantial disruption of the ability to conduct normal life functions.

Important medical events that may not meet the above criteria may be considered serious when, based upon appropriate medical judgement, they may jeopardize the subject and may require medical or surgical intervention to prevent one of the outcomes listed in this definition.

Serious adverse events (SAE) will include severe bleeding events (graded as a grade 3 or 4 on the WHO bleeding scale, see Table 1), and treatment-related serious

adverse events, including death, prolongation of existing hospitalization, or a persistent or significant disability/incapacity.

All SAEs will be followed through resolution or stabilization by a licensed study physician. All SAEs will be reviewed and evaluated and sent to the SMC for period review, and the IRB within 7 days of PI-notification, per VUMC IRB policy.

8.3 Reporting of SAE

Any AE that meets a protocol-defined criterion of an SAE will be reported per local IRB requirements, within 7 days of PI-notification, per VUMC IRB policy.

8.4 Recording of AEs/SAEs

Information regarding AEs and SAEs will be collected will be uploaded into a password-protected computerized database maintained within a secure, web-based application for building and managing online databases (REDCap) [see section 10].

8.5 Safety Monitoring Committee

Although the study poses no greater than minimal risk to participants, a safety monitoring committee (SMC) will be appointed to ensure the continuing safety of research participants, appropriateness of the study and to monitor termination criteria. The SMC will be comprised of three individuals with expertise relevant to the study, including pediatric medicine, biostatistics, and prior experience serving in this capacity. The SMC will be independent of the study team and will meet every six months. During these meetings, the SMC will review adherence to the protocol, reports of SAE and AE. The SMC will not perform any interim analyses but will be provided outcome data to inform safety decisions. The SMC will make recommendations regarding whether the study should continue unchanged or require modification/amendment. Based on the findings of the SMC reports, the study team may also elect to halt, suspend, or modify study.

The PI will be responsible for overseeing the study. They will be available at any time for questions from clinically relevant providers. They will also record adverse events and serious adverse events. Study-related SAE will be recorded in the study record and reported to the IRB within 7 calendar days of PI-notification. Summary reports will be submitted to the IRB annually and will contain a) the number of adverse events and b) the number of protocol violations and how each was handled.

9 Study Withdrawal/Discontinuation

Due to the study design, withdrawals are not anticipated. The primary focus of our study is to determine if a the EHR-based VTE risk prediction tool increases the number of high-risk patients reviewed by the VTE research team. Patients in both arms will continue to receive the current standard of care clinical practice for inpatient prophylactic anticoagulation. High-risk patients in the intervention arm will receive an additional chart review and subspecialty expertise.

10 Statistical Considerations

10.1 Statistical Analysis Plan

Primary analyses will be conducted at the encounter level, while secondary analyses will be conducted at the patient level (patients can potentially experience multiple encounters during the study period).

The rate of VTE events (primary outcome), the proportion of patients receiving prophylactic anticoagulation (secondary outcome), and the rate of bleeding-related adverse events (secondary outcome) will be compared between the intervention and control arms using unadjusted risk differences with 95% confidence intervals. We have not specified *a priori* any adjustment variables to include in multivariable models. Any variable with a clinically meaningful imbalance between the intervention and control groups will be adjusted for in multivariable regression models; given the large sample size we do not anticipate any such imbalances. For the primary outcome, a two-sided p-value (obtained from Pearson’s chi-square test) of < 0.05 will indicate statistical significance. Secondary subgroup analyses will be performed by patient gender, risk of VTE, primary diagnoses, surgical vs. nonsurgical, and consultants involved.

Primary analyses will be conducted by intention-to-treat; secondary analyses will be as treated, considering whether treatment recommendations were followed by the admitting team. Secondary as-treated analyses will be performed among the strata of high-risk patients (predicted probability $\geq$0.025) randomized to usual care, to the intervention but treatment recommendations were not followed, and to the intervention and treatment recommendations were followed.

10.2 Sample Size Considerations

At least 15,000 patients (~1 year of data), and no more than 45,000 patients (~3 years of data), will be randomized 1:1 to the intervention or control arms. If we assume an overall median risk of 1%, we will achieve 80% power to detect a risk reduction from

1% to 0.595% if there are at least 7500 subjects per group. We are aware that due to the low baseline event rate, we may need to enroll additional patients to achieve the necessary power. This will be the largest randomized controlled trial ever performed to answer this important question in pediatrics and will therefore be an important contribution to the literature both for pediatric hematology and in the field of pediatrics.

11 Privacy/Confidentiality Issues

At no time during the course of this study, its analysis, or its publication will patient identities be revealed in any manner. Patient privacy will be maintained by only using the minimum necessary data containing patient or provider identities. Identifiable data will be used for initial chart review and recommendations. As quickly as feasible, all data collected will be uploaded into a password-protected computerized database maintained within a secure, web-based application for building and managing online databases (REDCap) or stored on secure servers with user-level access control. The data will then be de-identified prior to the statistical analysis. All data will be reviewed as group summary information to minimize potential patient identification by the reviewing group. All identifiers will be removed prior to publication. The identifiable data will not be used in any other research projects and will be destroyed after six years following initial publication.

12 Follow-up and Record Retention

Enrollment for the study will last for a minimum of one year, although may run for up to three years in order to obtain sample size needed. For each participant, the study will commence at enrollment and study intervention will last until hospital discharge or in-hospital death. Patient clinical outcomes will be collected up until hospital discharge or death. Identified data in the secure database (REDcap) will be stored for up to three years following initial publication.

13 References

1. Raffini L, Huang YS, Witmer C, Feudtner C. Dramatic increase in venous thromboembolism in children's hospitals in the United States from 2001 to 2007. *Pediatrics*. 2009;124(4):1001-1008.
2. Monagle P, Cuello CA, Augustine C, Bonduel M, Brandão LR, Capman T, et al. American Society of Hematology 2018 Guidelines for management of venous thromboembolism: treatment of pediatric venous thromboembolism. *Blood Adv*. 2018;2(22):3292-3316.
3. Goudie A, Dynan L, Brady PW, Fieldston E, Brilli RJ, Walsh KE. Costs of Venous Thromboembolism, Catheter-Associated Urinary Tract Infection, and Pressure Ulcer. *Pediatrics*. 2015;136(3):432-439.
4. Kumar R, Rodriguez V, Matsumoto JM, Khan SP, Weaver AL, McBane RD, et al. Prevalence and risk factors for post thrombotic syndrome after deep vein thrombosis in children: a cohort study. *Thromb Res*. 2015;135(2):347-351.
5. Goldenberg NA. Long-term outcomes of venous thrombosis in children. *Curr Opin Hematol*. 2005;12(5):370-376.
6. Atchison CM, Arlikar S, Amankwah E, Ayala I, Barrett L, Branchford BR, et al. Development of a new risk score for hospital-associated venous thromboembolism in noncritically ill children: findings from a large single-institutional case-control study. *J Pediatr*. 2014;165(4):793-798.
7. Sharathkumar AA, Mahajerin A, Heidt L, Doerfer K, Heiny M, Vik T, et al. Risk-prediction tool for identifying hospitalized children with a predisposition for development of venous thromboembolism: Peds-Clot clinical Decision Rule. *J Thromb Haemost*. 2012;10(7):1326-1334.
8. Mahajerin A, Betensky M, Goldenberg NA. Thrombosis in Children: Approach to Anatomic Risks, Thrombophilia, Prevention, and Treatment. *Hematol Oncol Clin North Am*. 2019;33(3):439-453.
9. Mahajerin A, Jaffray J, Branchford B, Stillings A, Krava E, Young G, et al. Comparative validation study of risk assessment models for pediatric hospital-acquired venous thromboembolism. *J Thromb Haemost*. 2020;18(3):633-641.
10. Ellis HB, Sabatino MJ, Clarke Z, Dennis G, Fletcher AL, Wyatt CW, et al. The Importance of a Standardized Screening Tool to Identify Thromboembolic Risk Factors in Pediatric Lower Extremity Arthroscopy Patients. *J Am Acad Orthop Surg*. 2019;27(9):335-343.
11. Faustino EV, Hanson S, Spinella PC, Tucci M, O'Brien SH, Nunez AR, et al. A multinational study of thromboprophylaxis practice in critically ill children. *Crit Care Med*. 2014;42(5):1232-1240.
12. Raffini L, Trimarchi T, Beliveau J, Davis D. Thromboprophylaxis in a pediatric hospital: a patient-safety and quality-improvement initiative. *Pediatrics*. 2011;127(5):e1326-1332.
13. Stem J, Christensen A, Davis D, Raffini L. Safety of prophylactic anticoagulation at a pediatric hospital. *J Pediatr Hematol Oncol*. 2013;35(7):e287-291.
