# Supplementary material for: Model-Guided Decision-Making for Thromboprophylaxis and Hospital-Acquired Thromboembolic Events Among Hospitalized Children and Adolescents: The CLOT Randomized Clinical Trial
Source: JAMA Netw Open. 2023 Oct 13;6(10):e2337789. doi: 10.1001/jamanetworkopen.2023.37789 (PMC10576217; doi:10.1001/jamanetworkopen.2023.37789)
Supplement: Supplement 2. — eMethods 1. Details on Study Procedures eMethods 2. Details on Study Outcomes eTable 1. Confusion Matrix for the Prognostic Model at a Cut Point of 2.5% eTable 2. Type of Thromboembolism eTable 3. Characteristics of the Children and Adolescents Included in the CLOT Trial for Their First Admission During the Study Period eTable 4. Primary and Secondary Outcomes for All Randomized Patients for Their First Admission During the Study Period eTable 5. Reasons for Not Recommending Initiation of Pharmacologic Thromboprophylaxis to the Primary Team for Patients Randomized to the Intervention and With Elevated Risk for Developing Hospital-Acquired Venous Thromboembolism eTable 6. Reasons That the Primary Team Rejected the Recommendation for Initiating Pharmacologic Thromboprophylaxis for Patients Randomized to the Intervention and With Elevated Risk for Developing Hospital-Acquired Venous Thromboembolism eTable 7. Characteristics of the Children and Adolescents Included in the CLOT Trial with Elevated Risk for Developing Hospital-Acquired Venous Thromboembolism eFigure. Flow of Participants Through the CLOT Trial for Secondary As-Treated Analysis [file jamanetwopen-e2337789-s002.pdf]

Supplemental Online Content

Walker SC, French B, Moore RP, et al. Model-guided decision-making for thromboprophylaxis and hospital-acquired thromboembolic events among hospitalized children and adolescents. *JAMA Netw Open.* 2023;6(10):e2337789. doi:10.1001/jamanetworkopen.2023.37789

eMethods 1. Details on Study Procedures

eMethods 2. Details on Study Outcomes

eTable 1. Confusion Matrix for the Prognostic Model at a Cut Point of 2.5%

eTable 2. Type of Thromboembolism

eTable 3. Characteristics of the Children and Adolescents Included in the CLOT Trial for Their First Admission During the Study Period

eTable 4. Primary and Secondary Outcomes for All Randomized Patients for Their First Admission During the Study Period

eTable 5. Reasons for Not Recommending Initiation of Pharmacologic Thromboprophylaxis to the Primary Team for Patients Randomized to the Intervention and With Elevated Risk for Developing Hospital-Acquired Venous Thromboembolism

eTable 6. Reasons That the Primary Team Rejected the Recommendation for Initiating Pharmacologic Thromboprophylaxis for Patients Randomized to the Intervention and With Elevated Risk for Developing Hospital-Acquired Venous Thromboembolism

eTable 7. Characteristics of the Children and Adolescents Included in the CLOT Trial with Elevated Risk for Developing Hospital-Acquired Venous Thromboembolism

eFigure. Flow of Participants Through the CLOT Trial for Secondary As-Treated Analysis

This supplemental material has been provided by the authors to give readers additional information about their work.

© 2023 Walker SC et al. *JAMA Network Open.*

eMethods 1. Details on Study Procedures

All randomized patients had their predicted probability of hospital-acquired venous thromboembolism (HA-VTE) calculated upon admission and daily thereafter according to our previously developed and validated prognostic model for HA-VTE. The prognostic model is based on 11 variables that are automatically extracted from the electronic medical record (EMR). These variables are: patient age, whether the patient had a diagnosis of cancer, whether the patient had a history of thrombosis, whether the patient had placement of a central venous line during admission, whether the patient received surgery during admission, whether a blood gas was performed during admission, cardiology consultation or admission to the cardiology team, infectious disease consultation or admission to the infectious disease team, lactate, mean corpuscular hemoglobin concentration (MCHC), and red cell distribution width (RDW). The model provided excellent discriminatory ability in the derivation cohort (*C* statistic: 0.908; 95% CI: 0.896 to 0.918) and temporal external validation cohort (*C* statistic: 0.904; 95% CI: 0.894 to 0.913).

The data for the model variables were extracted from the Epic EMR into a Clarity report, which ran every day at midnight; thus, a patient's predicted probability of HA-VTE was updated as the clinical situation evolved. For calculation of the predicted probability of HA-VTE, unknown values for lactate, MCHC, and RDW were replaced with the median value in the derivation cohort: 1.3 mmol/L, 34.0 g/dL, and 14.3%, respectively.

For patients randomized to the intervention group, their predicted probability was displayed in the daily report for review by dedicated pediatric hematologists daily on weekdays. Patients at elevated risk on weekends and holidays were reviewed the following weekday if they remained at elevated risk.

Anticoagulation was recommended with prophylactic dosing of either a continuous infusion of unfractionated heparin (10u/kg) or q12h subcutaneous injections of enoxaparin (age and weight-based dosing: <3 months, 0.8mg/kg/dose; 3-12 months, 0.75 mg/kg/dose; 1-5 years, 0.6 mg/kg/dose; and >6 years, 0.5mg/kg/dose (max 40mg q12h)). No low molecular weight heparin levels were obtained to guide prophylactic dosing as part of the study.

Primary clinical teams were notified in person or via phone call by the study hematologists (APW, SCW) when a patient in the intervention group was reviewed and thromboprophylaxis was recommended. A brief note summarizing the recommendations was placed into the chart for all elevated risk patients who underwent hematology review as party of the study. Patients were reviewed once during a hospital admission and were not re-reviewed during that encounter.

© 2023 Walker SC et al. *JAMA Network Open.*

eMethods 2. Details on Study Outcomes

Throughout the study period, the radiology coordinator provided the research team with a monthly report of all inpatient pediatric radiology studies to identify patients with HA-VTE (e.g., extremity ultrasounds with Doppler and CT angiograms) completed in the prior month. The report was reviewed by the research team to identify patients with HA-VTE and the date of diagnosis. To ensure that all cases of HA-VTE were captured, a separate dataset was obtained after study closure from the EMR using ICD-9/10 codes for acute VTE. A subset of these was reviewed for accuracy, as in the prior model-development work.

© 2023 Walker SC et al. *JAMA Network Open.*

eTable 1. Confusion Matrix for the Prognostic Model at a Cut Point of 2.5%[a]

	No.		
	HA-VTE		
	Yes	No	Total
Predicted probability[b]			
≥2.5%	50	882	932
<2.5%	8	7770	7778
Total	58	8652	8710

Abbreviation: HA-VTE, hospital-acquired venous thromboembolism.

[a] Assessed among patients randomized to the control group.

[b] Predicted probability of hospital-acquired venous thromboembolism at admission.

Sensitivity: 0.86; 95% CI: 0.75 to 0.94
Specificity: 0.90; 95% CI: 0.89 to 0.90

© 2023 Walker SC et al. *JAMA Network Open.*

eTable 2. Type of Thromboembolism

	No.
Internal jugular, subclavian, or inferior vena caval thrombus	52
Extremity venous thrombus – central venous line associated	43
Arterial thrombus	8
Extremity venous thrombus – non-central venous line associated	6
Cerebral sinus venous thrombosis	5
Pulmonary embolism	3
Renal vein thrombus	2
Right arterial thrombus	2
Other	14
Total	135

© 2023 Walker SC et al. *JAMA Network Open.*

eTable 3. Characteristics of the Children and Adolescents Included in the CLOT Trial for Their First Admission During the Study Period

Characteristic	No. (%)[a]	
	Control group (n=7127)	Intervention group (n=7120)
Age, median (IQR), y[b]	1.0 (0, 10.1)	0.9 (0, 10.2)
Sex		
Female	3445 (48.3)	3343 (47.0)
Male	3682 (51.7)	3777 (53.0)
Race[c]		
Asian	185 (2.6)	166 (2.3)
Black	1098 (15.4)	1167 (16.4)
Multiple races	205 (2.9)	187 (2.6)
Other[d]	20 (0.3)	29 (0.4)
White	4803 (67.4)	4757 (66.8)
Unknown[e]	816 (11.4)	814 (11.4)
Ethnicity[c]		
Hispanic	1004 (14.1)	960 (13.5)
Non-Hispanic	5946 (83.4)	5964 (83.8)
Unknown[e]	177 (2.5)	196 (2.8)
Insurance type		
Government	4321 (60.6)	4309 (60.5)
Private	2705 (38.0)	2697 (37.9)
Other	22 (0.3)	14 (0.2)
Unknown[e]	79 (1.1)	100 (1.4)
History of cancer[b]	22 (0.3)	22 (0.3)
History of thrombosis[b]	8 (0.1)	2 (<0.1)
Admission type		
Emergency	2906 (40.8)	2900 (40.7)
Newborn	2514 (35.3)	2496 (35.1)
Elective	957 (13.4)	926 (13.0)
Urgent	557 (7.8)	609 (8.6)
Trauma	189 (2.7)	186 (2.6)
Unknown[e]	4 (0.1%)	3 (<0.1)
Weekend or holiday admission[f]	2683 (37.6)	2699 (37.9)
Placement of a central line during admission[b]	656 (9.2)	659 (9.3)
Receipt of surgery during admission[b]	1847 (25.9)	1837 (25.8)
Blood gas performed during admission[b]	1302 (18.3)	1261 (17.7)
Cardiology consulted during admission[b]	797 (11.2)	765 (10.7)
Infectious disease consulted during admission[b]	731 (10.3)	754 (10.6)
Earliest lactate during admission, median (IQR), mmol/L[b,g]	1.6 (1.0, 2.6)	1.6 (1.0, 2.7)
Earliest MCHC during admission, median (IQR), g/dL[b,h]	33.7 (32.8, 34.6)	33.7 (32.8, 34.6)
Earliest RDW during admission, median (IQR), %[b,i]	13.9 (12.6, 16.2)	13.8 (12.7, 16.1)
Predicted probability of HA-VTE at admission, median (IQR), %	0.2 (0.1, 0.4)	0.2 (0.1, 0.4)
Length of hospital stay, median (IQR), d	2.5 (1.8, 4.8)	2.5 (1.8, 4.8)

Abbreviations: CLOT, Children's Likelihood of Thrombosis; HA-VTE, hospital-acquired venous thromboembolism; IQR, inter-quartile range; MCHC, mean corpuscular hemoglobin concentration; RDW, red blood cell distribution width.

[a] Unless otherwise indicated.

[b] Included as an input to the prognostic model for hospital-acquired venous thromboembolism.

[c] Reported by patients and recorded in the electronic medical record.

[d] Includes American Indian and Native Hawaiian.

[e] Not recorded in electronic medical record.

[f] Weekend admission defined as admission occurring at any time on a Friday, Saturday, or Sunday. Non-weekend holidays were Christmas, Thanksgiving, and New Year's Day.

© 2023 Walker SC et al. *JAMA Network Open.*

[g] Unknown for 10,228 (71.8%) patients. For calculation of the predicted probability of hospital-acquired venous thromboembolism, unknown values were replaced with the value 1.3 mmol/L.
[h] Unknown for 5146 (36.1%) patients. For calculation of the predicted probability of hospital-acquired venous thromboembolism, unknown values were replaced with the value 34.0 g/dL.
[i] Unknown for 5153 (36.2%) patients. For calculation of the predicted probability of hospital-acquired venous thromboembolism, unknown values were replaced with the value 14.3%.

© 2023 Walker SC et al. *JAMA Network Open.*

eTable 4. Primary and Secondary Outcomes for All Randomized Patients for Their First Admission During the Study Period

	No. (%)		Risk difference per 1000 patients (95% CI)
	Control group (n=7127)	Intervention group (n=7120)	
Primary outcome			
HA-VTE	42 (0.6)	52 (0.7)	1.4 (-1.2, 4.1)
Secondary outcome			
Prophylactic anticoagulation	124 (1.7)	167 (2.3)	6.1 (1.4, 10.7)
Post-hoc outcomes			
All-cause in-hospital mortality	115 (1.6)	94 (1.3)	-2.9 (-6.9, 1.0)
Died with a VTE	7 (0.1)	7 (0.1)	0.0 (-1.0, 1.0)
30-day readmission	391 (5.5)	372 (5.2)	-2.6 (-10.0, 4.8)

Abbreviations: CI, confidence interval; HA-VTE, hospital-acquired venous thromboembolism.

© 2023 Walker SC et al. *JAMA Network Open.*

eTable 5. Reasons for Not Recommending Initiation of Pharmacologic Thromboprophylaxis to the Primary Team for Patients Randomized to the Intervention and With Elevated Risk for Developing Hospital-Acquired Venous Thromboembolism

Reason	No.
Prematurity (<34 weeks)	47
Clinically significant hemorrhage	41
Thrombocytopenia	39
Anticipated length of stay <72 hours	24
Clinical instability	15
Upcoming procedure	10
Coagulopathy	9
Severe renal dysfunction	8
History of significant hemorrhage	4
No change in clinical status	3
Pediatric hematology/oncology service recommended against thromboprophylaxis	3
Likely central line removal within 24 hours	3
Recent stroke	2
Recent neurosurgical procedure	2
Severe bleeding disorder	1
Imminent patient discharge	1
Total	212

Abbreviation: VTE, venous thromboembolism.

© 2023 Walker SC et al. *JAMA Network Open.*

eTable 6. Reasons That the Primary Team Rejected the Recommendation for Initiating Pharmacologic Thromboprophylaxis for Patients Randomized to the Intervention With Elevated Risk for Developing Hospital-Acquired Venous Thromboembolism[a]

Reason	No.
Physician preference	119
Imminent patient discharge	46
Likely central line removal within 24 hours	33
Patient's family declined	8
Consulting subspecialist team declined[b]	7
Upcoming procedure	5
Not consistent with goals of care (e.g., hospice)	4
Patient developed VTE between research team review and discussion with primary team	2
Cost	1
Total	225

Abbreviation: VTE, venous thromboembolism.

[a] Elevated risk defined as a predicted probability of developing hospital-acquired venous thromboembolism $\geq$2.5%.

[b] Consulting subspecialists included cardic transplant, liver, stem cell transplant, renal transplant, and neurosurgery.

© 2023 Walker SC et al. *JAMA Network Open.*

eTable 7. Characteristics of the Children and Adolescents Included in the CLOT Trial with Elevated Risk for Developing Hospital-Acquired Venous Thromboembolism[a]

Characteristic	No. (%)[b] Control group (n=932)	Intervention group, recommended and not initiated [c] (n=213)	Intervention group, recommended and initiated[d] (n=74)
Age, median (IQR), y[e]	4.5 (0.1, 14.3)	7.1 (0.5, 14.7)	2.2 (0.3, 14.3)
Sex			
Female	408 (43.8)	104 (48.8)	43 (58.1)
Male	524 (56.2)	109 (51.2)	31 (41.9)
Race[f]			
Asian	21 (2.3)	10 (4.7)	2 (2.7)
Black	165 (17.7)	24 (11.3)	12 (16.2)
Multiple races	26 (2.8)	5 (2.3)	4 (5.4)
Other[g]	6 (0.6)	3 (1.4)	1 (1.4)
White	622 (66.7)	148 (69.5)	47 (63.5)
Unknown[h]	92 (9.9)	23 (10.8)	8 (10.8)
Ethnicity[f]			
Hispanic	140 (15.0)	30 (14.1)	11 (14.9)
Non-Hispanic	785 (84.2)	181 (85.0)	63 (85.1)
Unknown[h]	7 (0.8)	0	2 (0.9)
Insurance type			
Government	610 (65.5)	134 (62.9)	47 (63.5)
Private	314 (33.7)	78 (36.6)	27 (36.5)
Other	1 (0.1%)	0	0
Unknown[h]	7 (0.8)	1 (0.5)	0
History of cancer[e]	47 (5.0)	15 (7.0)	5 (6.8)
History of thrombosis[e]	8 (0.9)	0	1 (1.4)
Admission type			
Emergency	366 (39.3)	78 (36.6)	32 (43.2)
Newborn	150 (16.1)	19 (8.9)	5 (6.8)
Elective	242 (26.0)	85 (39.9)	19 (25.7)
Urgent	152 (16.3)	31 (14.6)	16 (21.6)
Trauma	20 (2.1)	0	2 (2.7)
Unknown[h]	2 (0.2)	0	0
Weekend or holiday admission[i]	291 (31.2)	56 (26.3)	22 (29.7)
Placement of a central line during admission[e]	598 (64.2)	122 (57.3)	57 (77.0)
Receipt of surgery during admission[e]	569 (61.1)	141 (66.2)	53 (71.6)
Blood gas performed during admission[e]	637 (68.3)	134 (62.9)	58 (78.4)

© 2023 Walker SC et al. *JAMA Network Open.*

Cardiology consulted during admission[e]	432 (46.4)	100 (46.9)	36 (48.6)
Infectious disease consulted during admission[e]	424 (45.5)	97 (45.5)	40 (54.1)
Earliest lactate during admission, median (IQR), mmol/L[e,j]	1.7 (1.1, 2.8)	1.6 (0.9, 2.4)	1.6 (0.9, 2.7)
Earliest MCHC during admission, median (IQR), g/dL[e,k]	33.3 (32.4, 34.4)	33.3 (32.3, 34.4)	33.1 (32.1, 34.1)
Earliest RDW during admission, median (IQR), %[e,l]	15.0 (13.2, 17.5)	14.6 (13.1, 16.9)	14.9 (13.8, 17.8)
Predicted probability of HA-VTE at admission, median (IQR), %	2.5 (0.7, 4.4)	2.8 (1.0, 5.4)	3.2 (0.8,8.1)
Highest predicted probability of HA-VTE prior to diagnosis, median (IQR), %	6.3 (3.4, 11.2)	7.9 (4.4, 11.8)	9.0 (6.1, 14.7)
Length of hospital stay, median (IQR), d	12.8 (5.0, 37.1)	9.1 (4.9, 26.0)	16.9 (7.9, 27.9)

Abbreviations: CLOT, Children's Likelihood of Thrombosis; HA-VTE, hospital-acquired venous thromboembolism; IQR, inter-quartile range; MCHC, mean corpuscular hemoglobin concentration; RDW, red blood cell distribution width.

[a] Elevated risk defined as a predicted probability of developing hospital-acquired venous thromboembolism ≥2.5%.

[b] Unless otherwise indicated.

[c] Patients randomized to the intervention group and treatment was recommended, but treatment recommendations were not followed.

[d] Patients randomized to the intervention group, treatment was recommended, and treatment recommendations were followed.

[e] Included as an input to the prognostic model for hospital-acquired venous thromboembolism.

[f] Reported by patients and recorded in the electronic medical record.

[g] Includes American Indian and Native Hawaiian.

[h] Not recorded in electronic medical record.

[i] Weekend admission defined as admission occurring at any time on a Friday, Saturday, or Sunday. Non-weekend holidays were Christmas, Thanksgiving, and New Year's Day.

[j] Unknown for 212 (17.4%) patients. For calculation of the predicted probability of hospital-acquired venous thromboembolism, unknown values were replaced with the value 1.3 mmol/L.

[k] Unknown for 11 (0.9%) patients. For calculation of the predicted probability of hospital-acquired venous thromboembolism, unknown values were replaced with the value 34.0 g/dL.

[l] Unknown for 10 (0.8%) patients. For calculation of the predicted probability of hospital-acquired venous thromboembolism, unknown values were replaced with the value 14.3%.

© 2023 Walker SC et al. *JAMA Network Open.*

eFigure 1. Flow of Participants Through the CLOT Trial for Secondary As-Treated Analysis

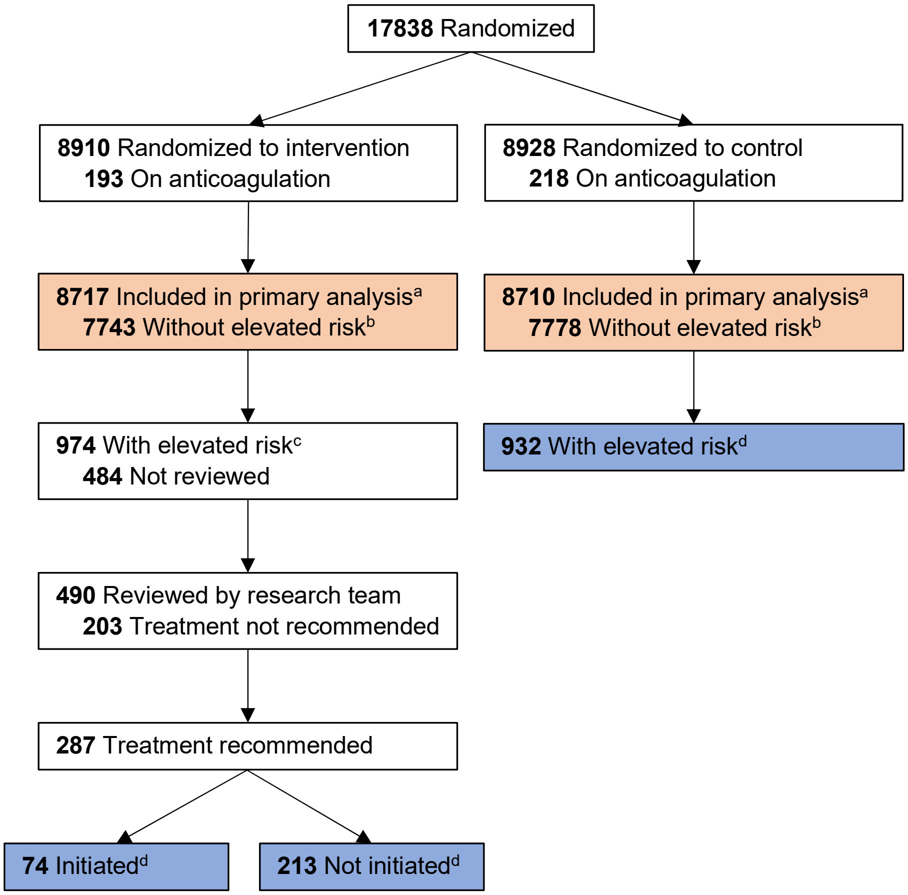

[a] Groups analyzed as part of the primary modified intention-to-treat analysis.
[b] Elevated risk defined as a predicted probability of developing hospital-acquired venous thromboembolism $\geq$2.5% at any point prior to a diagnosis of hospital-acquired venous thromboembolism.
[c] Includes 6 patients with a predicted probability <2.5% who were judged to be at elevated risk due to other reasons to be considered at risk for developing a hospital-acquired venous thromboembolism.
[d] Groups analyzed as part of the secondary as-treated analysis.

© 2023 Walker SC et al. *JAMA Network Open.*
