# Supplementary material for: Model-Guided Decision-Making for Thromboprophylaxis and Hospital-Acquired Thromboembolic Events Among Hospitalized Children and Adolescents: The CLOT Randomized Clinical Trial
Source: JAMA Netw Open. 2023 Oct 13;6(10):e2337789. doi: 10.1001/jamanetworkopen.2023.37789 (PMC10576217; doi:10.1001/jamanetworkopen.2023.37789)
Supplement: Supplement 3. — Data Sharing Statement [file jamanetwopen-e2337789-s003.pdf]

Data Sharing Statement

Walker. Model-Guided Decision-Making for Thromboprophylaxis and Hospital-Acquired Thromboembolic Events Among Hospitalized Children and Adolescents. *JAMA Netw Open*. Published October 13, 2023. doi:10.1001/jamanetworkopen.2023.37789

Data
Data available: No

Additional Information
Explanation for why data not available: Due to the inclusion of protected health information in the dataset, it will not be made publicly available. We would be happy to discuss potential collaboration with interested researchers.
